# Assessing Parkinson’s Rest Tremor from the Wrist with Accelerometry and Gyroscope Signals in Patients with Deep Brain Stimulation: An Observational Study

**DOI:** 10.3390/jcm14062073

**Published:** 2025-03-18

**Authors:** Martin Keba, Maie Bachmann, Jaanus Lass, Tõnu Rätsep

**Affiliations:** 1 Department of Neurology and Neurosurgery, Institute of Clinical Medicine, University of Tartu, 50406 Tartu, Estonia; tonu.ratsep@kliinikum.ee; 2 Department of Neurosurgery, North Estonia Medical Centre, 13419 Tallinn, Estonia; 3 Biosignal Processing Laboratory, Tallinn University of Technology, 19086 Tallinn, Estonia; maie.bachmann@taltech.ee (M.B.); jaanus.lass@taltech.ee (J.L.); 4 Department of Neurosurgery, Tartu University Hospital, 50406 Tartu, Estonia

**Keywords:** wearable device, tremor, accelerometry, gyroscope, inertial measurement unit, Parkinson’s disease, deep brain stimulation, tremor rating scale

## Abstract

**Background:** Wearable sensors are mainly used in Parkinson’s disease (PD) to assess motor symptoms and to aid clinicians in patient management. Inertial measurement units that simultaneously register accelerometric and gyroscope signals have been one of the most studied and practicable methods. The heterogeneity of described methods and clinical settings studied can discourage wearable device use and highlight the need for standardization. This study compares previously proposed accelerometry and gyroscope signal features for tremor assessment measured at the wrist. **Methods**: An inertial measurement unit registered accelerometry and gyroscope signals at the wrist from 18 PD patients treated with deep brain stimulation (DBS). Measurements were made in DBS on and off states. Signal features for both accelerometry and gyroscope were calculated—mean linear acceleration, mean angular velocity, root mean square, maximal amplitude and power of the 3–7 Hz frequency band. The outcome features were log-transformed and correlated to the Movement Disorder Society Unified Parkinson’s Disease Rating Scale (MDS-UPDRS) item 3.17 using linear regression. Intraclass correlation coefficient (ICC) values were calculated for the signal features. **Results**: A total of 108 tremor episodes were investigated. All signal features exhibited a strong correlation with the MDS-UPDRS tremor amplitude scale. Tremor ratings showed a stronger correlation with accelerometry (r = 0.964–0.970) than with gyroscope-derived features (r = 0.942–0.956). The best-performing feature was the mean linear acceleration (r = 0.970, R^2^ = 0.940), which also showed high reliability (ICC = 0.921). **Conclusions**: Different accelerometry and gyroscope signal features are viable in characterizing rest tremor at the wrist. Simpler accelerometry signal features can be preferred in conducting the MDS-UPDRS item 3.17 examination in PD patients with DBS using a wrist-worn inertial measurement unit. Future research to expand the validity and usefulness of wearable technologies in PD is warranted.

## 1. Introduction

Tremor is a cardinal symptom of Parkinson’s disease (PD), having a profound impact on life quality [1]. Precise, constant and convenient tremor assessment is becoming more important as patient monitoring technologies and advanced therapies evolve rapidly. Instrumental methods to assess PD tremor have been developed and wearable sensors like inertial measurement units (IMUs) that utilize accelerometry and gyroscope measurements are one of the most researched and perspective methods [2,3]. However, symptom reporting is mainly performed using tremor rating scales. The most widely used clinical rating scale for PD is the Movement Disorder Society Unified Parkinson’s Disease Rating Scale (MDS-UPDRS). In this scale, rest tremor amplitude is reported in item 3.17, where the examiner visually evaluates tremor on an ordinal scale ranging from 0 to 4 [4]. There are formidable downsides to using clinical tremor rating scales—low resolution, poor temporal contingency and reliability issues between raters and within subjects [5,6]. Clinical management of PD tremor is particularly challenging due to fluctuations over time, psychological modulation and the impact on quality of life. However, clinical scales may fail to capture all these relevant aspects [2]. Clinicians also rely on patients’ diaries or recollections to estimate the disease burden in the home setting. The subjective nature and recall bias hinder this approach and may not suffice for consequential treatment decisions. Moreover, patients with declined cognition or depression may fail to recognize and report their symptoms [1].

Wearable devices have intrinsic properties that overcome some of the limitations seen with clinical rating scales. Reporting digital outcomes allows objective and quantitative tremor assessment with high magnification. Good repeatability and low operator dependence has been shown for simple devices. This can make tremor assessment reliable and help clinically less experienced raters. The main advantage is that wearable sensors can be deployed at all times providing long-term monitoring. This helps clinicians understand daily fluctuations and irregularities and reveal potential treatment gaps [2,3].

On the other hand, wearable devices have several drawbacks. There is no consensus or a golden standard on the best method to register PD tremors. Heterogeneity derives from the different sensors used, measurement locations, signal processing and outcome calculations. Most of the reported studies utilize custom applications with closed-source programming. Also, tremor measurements have been performed in different clinical settings, such as ambulatory symptom monitoring, task-specific severity tests, research endpoints and diagnostic aids. Previous research has shown different methods and opportunities, but to date wearable devices have not been transformative for day-to-day neurological practice [2,3]. Different scenarios require validation, and the best-performing method in one situation may not perform well in another. Furthermore, the cost-effectiveness, data management issues and cybersecurity risks are unknown [7].

Deep brain stimulation (DBS) is one of the most effective treatment options to alleviate tremor in advanced PD [8]. Several DBS programming sessions are often undertaken to obtain the best treatment response and a good side effect profile [9]. An individualized and up-to-date DBS therapy is valuable because, to an extent, the stimulation can readjust to the dynamic and progressive nature of the disease [8,9]. Inputs from wearable devices can be used in adaptive DBS solutions [10]. As improvements in DBS hardware provide more opportunities, the granularity of symptom assessment with wearable sensors can help reduce the optimization burden in DBS programming. For example, algorithm-guided programming has shown promising results in automating the monopolar review. This would lead to reduced time spent programming DBS and less experienced clinicians could achieve better results [11].

This observational study investigates PD tremor in DBS patients with accelerometric and gyroscopic signals using an IMU at the wrist. A selection of previously published signal features is assessed alongside the clinical MDS-UPDRS rest tremor amplitude scale. This study illustrates how the PD clinical tremor assessment can be supported with a wearable device and aims to identify the signal features that perform the best.

## 2. Materials and Methods

### 2.1. Study Participants

Idiopathic PD patients with prior DBS surgery undergoing reprogramming sessions at Tartu University Hospital, Estonia, were enrolled. Patients were eligible if they had a rest tremor determined by the MDS-UPDRS item 3.17 (Table 1) of one point or more in the upper extremity in the DBS-off/Dopa-on state [4]. Subjects were enrolled from January 2023 to August 2024. Written consent was obtained. The study was approved by the Ethics Committee on Human Research of the University of Tartu (Number 372/T-3) and complied with the Declaration of Helsinki.

### 2.2. Clinical Assessments and Data Collection

Patients were examined after DBS reprogramming with optimal stimulation settings. Clinical evaluations were conducted in four states depending on DBS and dopaminergic therapy. The interval for clinical re-evaluation between different states was 1 h, but if the patient expressed discomfort owing to the lack of active therapy, the clinical assessments were made consecutively. Alongside the motor exam, other parts of the MDS-UPDRS were assessed. Additionally, the 39-Item Parkinson’s Disease Questionnaire (PDQ-39) and the Montreal Cognitive Assessment test (MoCa) were reported [12,13].

### 2.3. Device Descriptions

**Figure 1:**
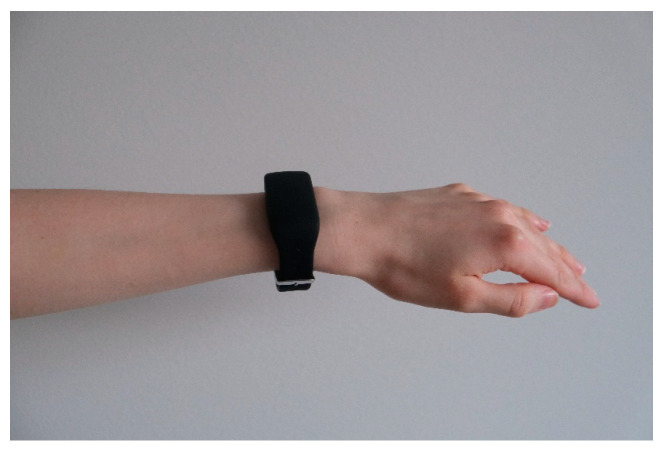
Inertial measurement unit placement at the wrist.

Tremor measurements were made using a 6 degrees of freedom IMU, registering linear acceleration in three dimensions and angular velocity in three rotational axes (Axivity, Newcastle upon Tyne, UK). The IMU was housed in a rubber wristband and worn proximal to the wrist joint (Figure 1). Acceleration and angular velocity signals were recorded at sampling rate of 100 Hz.

### 2.4. Measurement Protocol

Rest tremor was measured from the more affected arm in the morning before dopaminergic therapy had been administered and under optimal DBS parameters. After measurements in the DBS-on/Dopa-off state, stimulation was turned off and measurements were remade depending on patient discomfort from 5 min to half an hour in the DBS-off/Dopa-off state. During this time, the change in rigidity and bradykinesia was determined clinically and the stability of rest tremor was visually verified. Patients were seated with forearms resting on the arms of the chair, allowing the hand to move freely [4,6]. The subjects were instructed to relax and not to make any voluntary movements. Episodes with dyskinesias and voluntary movements that interfered with accurate tremor measurements were discarded. Clinical notations and timestamps were made regarding tremor amplitude and presence. Each recording lasted for at least 1 min.

### 2.5. Signal Preprocessing and Feature Extraction

The linear acceleration signals were transformed from gravity units to cm/s^2^. Raw accelerometry and angular velocity signals were filtered using a 4th-order Butterworth bandpass filter of 1–40 Hz to remove low-frequency noise and fast oscillations unrelated to the tremor. Signal filtering settings were chosen to complement both acceleration and gyroscope signals, as recommended in previous research [14,15]. The filtered signal was visually inspected, and three distinct 10 s epochs without artefacts were selected for further analysis. As the acceleration and angular velocity axes were perpendicular, resultant vectors for sequential feature analysis were calculated as shown in (1) [15,16].

(1) $$a=\sqrt{x^{2}+y^{2}+z^{2}};\;\omega =\sqrt{\omega_{p}^{2}+\omega_{r}^{2}+\omega_{y}^{2}},$$

The mean linear acceleration (ACC) and mean angular velocity (AV) were calculated as the sum of the resultant acceleration or angular velocity divided by the number of samples in the 10-s epoch [14,17]. Root mean square values were calculated in a 1 s moving window for the accelerometry (RMS-a) and gyroscope (RMS-g) signal [17,18].

The signal power of the 3–7 Hz frequency band and the maximal tremor amplitude were calculated as described by Smid [19]. The Welch’s power spectral density was estimated from the accelerometry and gyroscope signal to calculate the power of the typical rest tremor in the 3–7 Hz frequency band. A window width of 1 s and a 50% overlap were chosen. From the resulting power spectrum periodograms, the area under the curve (AUC) in the 3–7 Hz frequency band was calculated for the acceleration (AUC-a) and gyroscope (AUC-g) using trapezoidal numerical integration [14,19]. The maximal tremor amplitude (AMP) was calculated from the accelerometry signal by consecutive integration. First, signal velocity was determined by an approximate cumulative numerical integral with the trapezoidal method. The mean velocity was subtracted from the signal to correct for the added constant during the integration step. Then, the corrected velocity was numerically integrated to calculate the signal displacement. This vector was further filtered using a non-causal second-order 1.2 Hz high-pass Butterworth filter to suppress the drift caused by integration. The maximal amplitude was determined as the highest peak in the absolute displacement vector and was multiplied by two [19]. Signal processing, analysis and feature extractions were performed in MATLAB version R2022b (MathWorks, Natick, MA, USA).

### 2.6. Statistical Analysis

Descriptive statistics were applied to summarize the study population, using means when the data met the normality requirement and medians otherwise. The treatment effect of DBS in the Dopa-off state on rest tremor severity was assessed by the Wilcoxon signed-ranked test and for the total score of the MDS-UPDRS motor exam by the paired samples *t*-test. The measured signal features were checked for normal distribution with the Shapiro–Wilk test, showing a non-Gaussian distribution. According to the Weber–Fechner law of psychophysics, instrumental measurements and ordinal tremor rating scales follow a logarithmic relationship. This can be expressed as shown in (2), where tremor signal is denoted as TS and tremor rating scale as TRS [6].

(2) logTS=α×TRS+β

Taking this into consideration, the tremor signal values obtained from the three distinct epochs were averaged and then transformed to a logarithmic scale. The relationships with the tremor rating scale were assessed with linear regression. The test-retest reliability of the measurements was assessed by the intraclass correlation coefficient (ICC). A two-way mixed effects, single measurement, absolute agreement model was used. Values between 0.75 and 0.9 indicated good and values bigger than 0.9 were considered to have excellent reliability. A *p*-value of <0.05 was considered statistically significant. Statistical analysis was conducted in SPSS version 29 (International Business Machines Corporation, New York, NY, USA).

## 3. Results

Eighteen patients with PD treated with bilateral subthalamic nucleus stimulation were investigated. The demographics and clinical characteristics of the study group are presented in Table 2. In total, 108 distinct signal segments were analyzed from measurements made in the DBS-on and DBS-off states. The rest tremor amplitude scores, alongside the DBS-on or DBS-off status, are depicted in Table 3. The mean therapeutic effect between DBS-On/Dopa-Off and DBS-Off/Dopa-Off states in the MDS-UPDRS part III was 32.2 (95% CI [25.0–39.4], *p* < 0.05). The median decrease observed in the tremor amplitude scale was 3 points with the stimulation therapy (Z = 3.796, *p* < 0.05).

**Figure 2:**
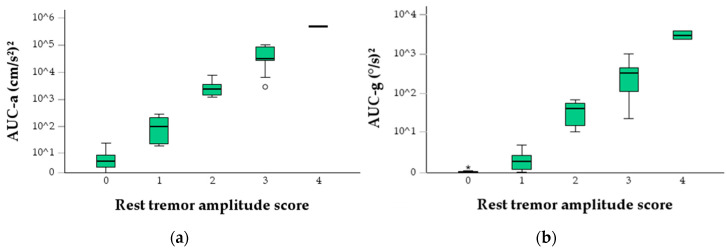

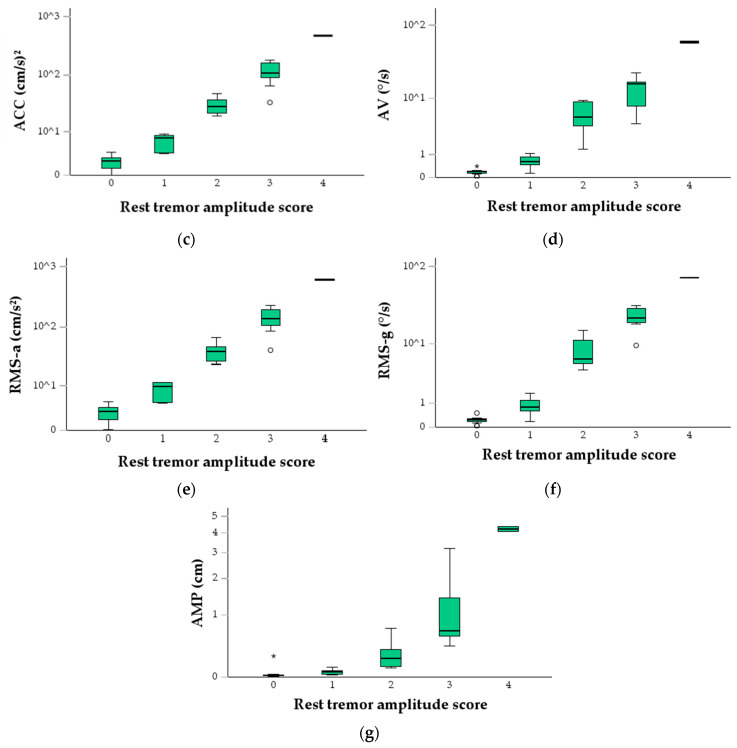
Boxplots of investigated signal features in a logarithmic scale shown across the MDS-UPDRS 3.17 rest tremor amplitude score—accelerometry AUC power of 3–7 Hz band (**a**), gyroscope AUC power of 3–7 Hz band (**b**), mean linear acceleration (**c**), mean angular velocity (**d**), accelerometry RMS (**e**), gyroscope RMS (**f**), maximal amplitude (**g**). Circles indicate mild outliers, representing values that deviate 1.5 times the interquartile range (IQR). Asterisks denote extreme outliers, marking values that exceed 3 times the IQR.

The log-transformed signal features showed a good linear correlation to the MDS-UPDRS 3.17 rest tremor score (Figure 2, Table 4). Accelerometry signal features outperformed their gyroscopic counterparts, having higher correlation and coefficients of determination values. The strongest correlation was seen with the mean linear acceleration (r = 0.970) and weakest with the maximal tremor amplitude (r = 0.919).

Intraclass correlation coefficients showed excellent reliability for the investigated signal features (Table 5).

## 4. Discussion

This study illustrates how different digital outcomes from accelerometric and gyroscopic signals can be used to measure PD rest tremor at the wrist. The investigated log-transformed features showed a significant linear relationship with the clinical tremor scale. The strongest correlation with the MDS-UPDRS item 3.17 was exhibited by the mean linear acceleration. Similarly to previous studies, the physically nearest parameter to the tremor scale, the maximal tremor amplitude, had comparatively lower correlation values in contrast to simpler signal features [14]. The maximal observed displacement in rest tremor is usually more evident at the fingers, making more proximal recording locations for amplitude assessment less sensitive [20].

We found that correlation coefficients were higher with signals derived from the accelerometer compared to their gyroscopic counterparts. This difference was modest, but present in pairwise assessment of mean acceleration, root mean square and signal power. Studies that have assessed rest tremor from finger-mounted IMUs have not shown a clear advantage of one over the other [18,21]. However, we are unaware of studies incorporating both methods for PD rest tremor assessment at the wrist in DBS patients. Accelerometers may have an advantage if the observed tremor has a greater linear movement but gyroscopes may have an advantage if it has greater rotational movement. For example, tremor in the head is more rotational, and therefore the gyroscopic assessment is more precise [22]. The typical pill-rolling movement associated with rest tremor is not as pronounced at the wrist and is not universally present in all PD patients [23]. Nevertheless, combining both modalities will offer an authentic representation of the underlying tremor and a set of signal features can exhibit higher correlation to tremor severity than single features.

Accelerometry measurements from the distal forearm may also be favorably influenced by external factors—the setting of the MDS-UPDRS item 3.17 and possible DBS effects on tremor. Tremor involving predominantly the fingers, as opposed to tremor with forearm pronation-supination or tremor with a dominant linear movement, can have different signal properties. Differences regarding tremor phenotypes could be further detailed with future research [24,25].

The investigated signal features were reliably reproducible, and the best ICC scores were comparable to or better than those of experienced PD tremor clinical assessors. For example, Richards and colleagues reported an ICC score of 0.84 for experienced clinicians [5]. Test-retest reliability values reported are comparable with similar research when tremor measurements are made within minutes [3].

Wearable devices should be easy to use and convenient for the patient and their preferences may differ from researchers’ [24]. Smartwatches offer a practical solution, as they already have a role in health monitoring and are widely used. Furthermore, modern smartwatches are capable of registering accelerometry and gyroscope signals with a sampling frequency of 100 Hz [26]. We made our measurements by an IMU sensor imitating a smartwatch as it was worn at the same position and used the same sampling frequency. The results of our study are useful for future application development and we have shown that non-customized IMUs can be easily utilized to support a routine clinical task.

Wearable device use is hindered by the heterogeneity of proposed methods and lack of standardization. The main strength of this study is that we have measured tremor amplitude in the MDS-UPDRS item 3.17 conditions and simultaneously registered accelerometry and gyroscope signals. Also, the selected signal features cover the most frequently used digital outcomes improving comparability. Further studies should be undertaken to increase the precision and validity of tremor measurements in different settings using relevant singular or multiple signal features and even multimodal signals. Commercial devices and associated applications will increase the availability of wearable devices, but open-source and independent research is still warranted [2].

Surface electromyograms (EMGs) have been also used at the forearm to assess PD tremor. EMGs can accurately depict rhythmic motor unit activity and detect tremor and the percentage of time that tremor is present. For tremor severity assessments, the estimates are not as precise as recorded from an IMU [27,28]. The main value of an EMG lies in its diagnostic utility as it can distinguish different types of tremor using frequency analysis, muscle activation patterns, coherence analysis and responses to external influences. IMUs can also reliably distinguish PD and essential tremor using the tremor stability index. This index has shown comparable diagnostic accuracy to radionucleotide studies that are used in early PD [27].

Ideally, wearable devices and applications for PD management should engage the whole spectrum of motor symptoms [20]. Incorporating measurements for dyskinesias, off-states, freezing of gait and falling increases functionality but can offset precision and validation efforts [24,29,30]. Ambulatory monitoring using IMUs is feasible and can provide the clinician valuable information that otherwise may be undetected. In addition to long-term symptom monitoring, wearable devices can aid in telemedicine, as the environment for tremor severity examination can be easily simulated [2,3]. PD patients with DBS may benefit from wearable technologies the most. Wearable devices could detect a digital threshold derived from machine learning algorithms, that could be correlated to the need to seek clinical consultations and reprogramming sessions [29,31]. As wearable devices and DBS therapies evolve, closed-loop solutions with adaptive stimulation become more promising [10]. Although some PD patients today may feel indifferent towards gadgets and technology, this trend is expected to shift with changing generations, as younger individuals are more accustomed to smart devices and digital healthcare solutions. From a healthcare management standpoint, wearable devices can optimize clinical treatment but increase the demand for data storage, security and utility [7,20,32].

This study has several limitations. First, the clinical tremor rating assessment was conducted by one rater (M.K.). Recognizing this shortcoming, the clinical rater had previously been involved in the patients’ PD management and has sufficient clinical experience. Second, the dataset was not balanced in tremor severity categories. This problem is often encountered in similar studies, due to the scarcity of severe tremors [19]. Third, selecting 10 s epochs for tremor analysis may be too long as it is prone to involuntary movements and temporal instability of tremor severity [16]. On the other hand, signal selection was based on visual inspection during the measurements and after signal preprocessing. This invokes a selection bias, but allows for a temporal correlation to the clinical tremor scale. Also, the sample size is relatively small, and our results are not confirmed in out-of-sample subjects. Finally, we did not investigate tremor with a comprehensive list of possible signal features, but reported a selection of outcomes that, in our view, have been studied the most.

## 5. Conclusions

Different PD tremor signals from a wrist-worn IMU can be used for tremor severity investigations. Accelerometry-derived signal features, for example the mean linear acceleration, may be preferred when conducting the MDS-UPDRS tremor amplitude assessment in PD patients with DBS. IMUs have shown clinical benefits and should be considered as an adjunct in PD management. Future large-scale studies are needed to increase the validity and practicality of wearable devices focusing on the community setting. This may enable their clinical potential in telemedicine and for personalized DBS therapy.

## Figures and Tables

**Table 1:** The MDS–UPDRS item 3.17. Rest tremor amplitude—upper extremity.

Tremor Grade	Visual Assessment
0: Normal	No tremor
1: Slight	<1 cm in maximal amplitude
2: Mild	≥1 cm but <3 cm in maximal amplitude
3: Moderate	≥3 cm but <10 cm in maximal amplitude
4: Severe	≥10 cm in maximal amplitude

**Table 2:** Patient demographics and clinical characteristics.

Variable		Value
Men (%)		15 (83.3)
Age (SD)		59.5 (7.3)
Age at diagnosis (SD)		47.9 (6.1)
Years from PD diagnosis (IQR)		9.5 (10.3)
Years to motor fluctuations (SD)		7.2 (3.8)
Years to dyskinesias (SD) *		9.4 (4.8)
Years to DBS operation (SD)		9.8 (4.9)
Levodopa daily dose, mg (SD)		694.4 (301.9)
Modified Hoehn–Yahr scale (IQR)	DBS-on/Dopa-on	1.5 (1.1)
Modified Hoehn–Yahr scale (IQR)	DBS-off/Dopa-off	2 (1.1)
PDQ-39SI (SD)		40 (16.8)
MoCa (SD)		27.1 (1.3)
MDS-UPDRS I (SD)		10.2 (5.8)
MDS-UPDRS II (SD)		14.5 (5.8)
MDS-UPDRS III (SD)	DBS-on/Dopa-on	16.9 (13.4)
	DBS-on/Dopa-off	24.5 (20.0)
	DBS-off/Dopa-on	40.8 (17.5)
	DBS-off/Dopa-off	56.7 (23.1)
MDS-UPDRS item 3.17 (IQR)	DBS-on/Dopa-off	0 (1)
	DBS-off/Dopa-off	3 (1)

*—dyskinesias were present in eight patients, PDQ-39SI—The 39-item Parkinson’s Disease Questionnaire summary index, MoCa—Montreal Cognitive Assessment test.

**Table 3:** MDS-UPDRS item 3.17.

Patient	1	2	3	4	5	6	7	8	9	10	11	12	13	14	15	16	17	18
DBS-off	3	2	3	2	3	2	3	1	3	2	2	3	2	3	3	3	4	4
DBS-on	0	0	1	1	0	0	0	0	2	0	0	1	0	0	0	1	1	2

**Table 4:** Summary of regression analyses.

TS	α (95% CI)	β (95% CI)	r	R^2^
AUC-a	1.304 (1.180, 1.429)	0.611 (0.355, 0.866)	0.964	0.930
AUC-g	1.281 (1.104, 1.459)	−1.401 (−1.764, −1.037)	0.929	0.864
ACC	0.579 (0.528, 0.630)	0.289 (0.185, 0.393)	0.970	0.940
AV	0.663 (0.581, 0.745)	−0.829 (−0.997, −0.661)	0.942	0.888
RMS-a	0.579 (0.526, 0.631)	0.398 (0.291, 0.505)	0.968	0.937
RMS-g	0.696 (0.622, 0.771)	−0.725 (−0.877, −0.573)	0.956	0.914
AMP	0.557 (0.474, 0.640)	−1.728 (−1.898, −1.558)	0.919	0.845

TS—tremor signal feature, r—Pearson correlation coefficient (*p* < 0.05 for all regression analyses), R^2^—coefficient of determination.

**Table 5:** Intraclass correlation values for the log-transformed signal features.

Feature	ICC	95% Confidence Interval	
		Lower	Upper
AUC-a	0.986	0.975	0.992
AUC-g	0.983	0.971	0.991
ACC	0.921	0.868	0.956
AV	0.906	0.844	0.947
RMS-a	0.919	0.863	0.955
RMS-g	0.901	0.837	0.945
AMP	0.903	0.840	0.946

ICC—intraclass correlation coefficient (two-way mixed effects, single measurement, absolute agreement).

## Data Availability

Data will be made available upon reasonable request.
